# Effects of Age on Intervertebral Disc Tissue Morphology and Gene Expression in the ADAM8-Inactivation Mouse

**DOI:** 10.3390/cells15080730

**Published:** 2026-04-20

**Authors:** Lutian Yao, Huan Wang, Zuozhen Tian, Frances S. Shofer, Ling Qin, Yejia Zhang

**Affiliations:** 1 Department of Orthopedic Surgery, Perelman School of Medicine, University of Pennsylvania, Philadelphia, PA 19104, USA; yaolutian@gmail.com (L.Y.); huan.wang@pennmedicine.upenn.edu (H.W.); qinling@pennmedicine.upenn.edu (L.Q.); 2 Department of Orthopedic Surgery, Tongji Hospital, Huazhong University of Science and Technology, Wuhan 430030, China; 3 Physical Medicine & Rehabilitation, Hospital of the University of Pennsylvania, Philadelphia, PA 19104, USA; zuozhen@pennmedicine.upenn.edu; 4 Department of Emergency Medicine, Perelman School of Medicine, the University of Pennsylvania, Philadelphia, PA 19104, USA; shofer@pennmedicine.upenn.edu; 5 Section of Rehabilitation Medicine, Corporal Michael J. Crescenz Veterans Affairs Medical Center, Philadelphia, PA 19104, USA

**Keywords:** ADAM8, inactivation, intervertebral disc, injury, age, inflammation

## Abstract

**Highlights:**

**What are the main findings?**

- Histological scores increased with aging in intact intervertebral discs (IVDs), and no differences were found between ADAM8-mutant and wild type (WT) mice.
- The injury-related differences in gene expression (mutant compared with WT) in young adult mice diminish with increasing age.

**What are the implications of the main findings?**

- Gene expression is a more sensitive measure of IVD responses to injury and differences between mutant and WT mice than histological features.
- To identify differences between WT and *Adam8^EQ^* mouse IVDs, 3-month-old mice are superior to older mice.

**Abstract:**

**Purpose**: To determine which age of mice should be used to compare the effects of ADAM8 mutation on intervertebral disc (IVD) responses to injury. **Methods**: IVDs of ADAM8 mutant (*Adam8^EQ^*) and wild type (WT) mice, aged 3, 10 and 18 months were injured. IVD tissues were harvested 1 week post injury for histological and molecular studies. **Results**: Histological scores increased with aging in intact IVDs, and there were no differences between *Adam8^EQ^* and WT mice (n = 11–28; *p* > 0.05). Safranin O-staining was less intense in 10-month than in 3-month-old mice, in both intact and injured IVDs (n = 3–15; *p* < 0.05). *Cxcl1*, *Il6*, and *Adam8* gene expression levels were higher in the injured tail IVDs of 3-month-old *Adam8^EQ^* than WT mice (n = 18–30; *p* < 0.05); the injury-related differences diminished with increasing age. **Conclusions**: No histological differences were found between *Adam8^EQ^* and WT mouse IVDs at 3, 10 or 18 months of age, in the intact or injured discs. The differences in inflammatory marker gene expression were detectable at age 3 months, but were less evident when the injury occurred at age 10 or 18 months. Therefore, to identify differences in injury responses between WT and *Adam8^EQ^* mouse IVDs, 3-month-old mice are superior to older mice.

## 1. Introduction

Back pain related to intervertebral disc (IVD) degeneration is common in the United States [1]. Elucidating the mechanisms of disease progression and developing targeted therapies to slow IVD degeneration and mitigate pain are essential for addressing this burden in an aging population. The present study aimed to determine which age of mice should be used to model IVD degeneration, since back pain is most prevalent in middle-aged humans [2]. The rationale to model IVD degeneration with young adult mice is that chronic back pain is frequently associated with a history of spinal injury [3], and persistent symptoms often are accompanied by radiological evidence of disc herniation and adjacent osseous pathology [4,5]. These clinical findings suggest that recurrent shear forces and mechanical insults to the spine, often start early in life, and may underlie the pathogenesis of back pain later [5].

Animal models with IVD injury offer valuable insight into the early cellular and molecular responses that occur in IVDs after injury [6,7], since human IVD tissue is not readily obtainable during early degeneration in back pain patients. Injury to the mouse IVD reliably produces molecular and histological changes associated with IVD degeneration [8,9,10,11], like those observed in humans [12].

Most studies of IVD degeneration-like changes in mice to date have used young adult animals [8,9,10,11]. A relevant issue is whether using older mice may more faithfully recapitulate human disease, given its peak prevalence in middle age [2]. Mouse and human ages are not directly equivalent, but general ranges allow comparison: 3–6 months (young adult mice), 10–15 months (middle-aged), and 18–24 months (elderly) [13]. Beyond 24 months, survivorship of mice declines sharply. Multiple studies have described spontaneous disc degeneration in aging mice [14,15,16]. A recent study has shown that aging has relatively minor effects on IVD response to injury in wild type (WT) mice [17], but the effects of gene mutations on responses to disc injury have not been described previously.

ADAM8 cleaves fibronectin in degenerative human IVDs [18]. The resulting 30 kDa fibronectin fragment has catabolic effects on the disc extracellular matrix [19]. To investigate the significance of ADAM8 proteolytic activity for cartilage integrity, an inactivation mutant (*Adam8^EQ^*) was created by substituting Glu^330^ with Gln, a modification that blocks enzyme self-activation [20]. Mice with this mutation develop normally and exhibit a milder form of collagen-induced arthritis compared to WT mice [20]. Previous studies showed that *Adam8^EQ^* mice had thicker annulus fibrosus (AF) than WT controls at 10 months of age in the lumbar spine IVDs [21]. In the injured tail discs, *Adam8^EQ^* mice had higher expression of inflammatory marker [e.g., Interleukin (*Il*) *6*, *Cxcl1*] genes than WT mice at 3 months of age [22]. *Il6* gene expression marks early inflammatory response, and *Cxcl1* gene expression is elevated at least until 4 weeks post IVD injury [23]. *Adam8* gene expression was also included in the panel of markers, because the ADAM8 protein level is elevated in degenerative IVDs; ADAM8 cleaves fibronectin, producing catabolic fibronectin fragments [18], thereby initiating the extracellular matrix degradation cascade [19].

ADAM8 is a versatile player in cancer cell migration, mechanics, and extracellular matrix remodeling [24]. However, ADAM8 mutation resulting in inactivation of the enzyme has not been described in humans. The mouse model with ADAM8 inactivated has been used in the current study to determine the effects of this enzyme on IVD degeneration, in intact tissues with age and in response to injury. The results will inform if inhibition of ADAM8, an enzyme responsible for generating the catabolic fibronectin fragment, would delay or prevent IVD degeneration.

The present study aimed to determine whether age influences the IVD injury response in ADAM8-mutant mice. Histological features, along with inflammatory markers and extracellular matrix gene expression in the context of injuries, and time points post injuries have been selected based on previous studies [17,23]. To determine what age of mice is most appropriate for comparison of injury responses between the mutant and WT mice, histological features and gene expression 1 week after disc injury in *Adam8^EQ^* mice aged 3, 10, and 18 months were compared with age-matched controls.

## 2. Materials and Methods

**Mice.** All animal experimental procedures were approved by the Institutional Animal Care and Use Committee of the University of Pennsylvania (approval #: 805511), and all methods were performed in accordance with the relevant guidelines and regulations, in compliance with the Animal Research: Reporting of In Vivo Experiments (ARRIVE) guidelines. A breeding pair of *Adam8^EQ^* mice, a generous gift from Dr. AnneMarie Malfait, was transported from Rush University Medical Center to the University of Pennsylvania. A breeding pair of DBA/1LacJ mice (the Jackson Laboratory, Bar Harbor, ME, USA) was used to produce WT control mice to match the genetic background of the *Adam8^EQ^* mice. All mice used were bred and housed under pathogen-free conditions in the same facility, as described previously [23]. At the endpoints in the experiments, individual animals were placed into an empty euthanasia chamber, which was filled gradually with CO_2_ gas. Death of the animal was verified by cessation of cardiovascular and respiratory activity, body movements, and lack of corneal reflex. 149 mice were used in this study (77 *Adam8^EQ^* mice; 3-month-old: 30; 10-month-old: 29; 18-month-old: 18; 72 WT mice; 3-month-old: 38; 10-month-old: 16; 18-month-old: 18). Both male and female mice were included.

**Figure 1:**
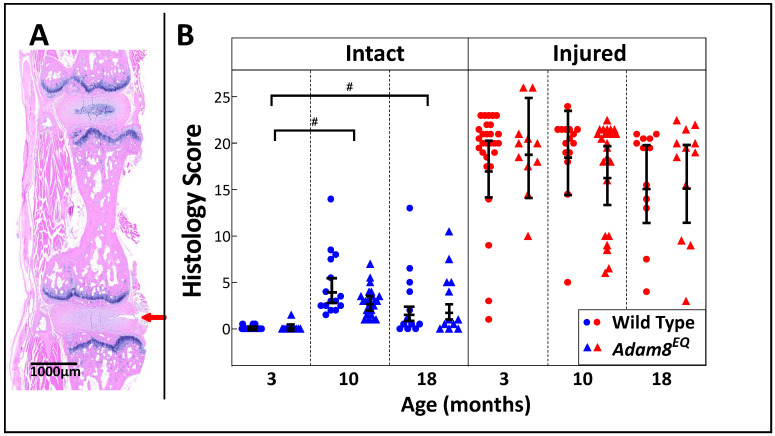
**Histological Scores of Intact and Injured Intervertebral Discs of Wild Type and ADAM8-Mutant (*Adam8^EQ^*) Mice at Age 3-, 10-, or 18-months**. (**A**) H&E-stained section of intact and adjacent injured discs from a 10-month-old *Adam8^EQ^* mouse; **red arrow**: the direction of injury; **Bar**: 1000 µm; (**B**) Histological score; each symbol represents score of one mouse [at 3 months: WT (n = 28), *Adam8^EQ^* (n = 11); 10 months: WT (n = 15), *Adam8^EQ^* (n = 24); 18 months: WT (n = 12), *Adam8^EQ^* (n = 12)]. **Error Bar**: mean ± 95% confidence interval; ^#^ *p* < 0.01.

**Figure 2:**
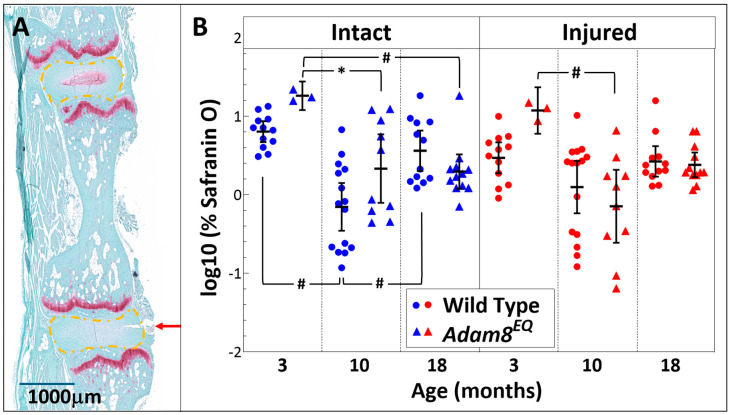
**Safranin O Staining in the Intervertebral Discs of Wild Type (WT) and ADAM8-mutant (*Adam8^EQ^*) Mice**. (**A**) intact and adjacent injured discs from a 10-month-old *Adam8^EQ^* mouse, stained with safranin O; **red arrow** points to the direction of injury; **orange** circled areas were quantified; **bar**: 1000 µm (**B**) pixel proportion of safranin O (red) staining reflecting mucopolysaccharide content in the disc. Each symbol represents score of one mouse [3 months: WT (n = 12), *Adam8^EQ^* (n = 3); 10 months: WT (n = 15), *Adam8^EQ^* (n = 10); 18 months: WT (n = 12), *Adam8^EQ^* (n = 12)]; **error bar**: mean ± 95% confidence interval; ^#^ *p* < 0.01; * *p* < 0.05.

**Tail injury surgery.** Mice were anesthetized in an aseptic setting, with an intraperitoneal injection of ketamine-Xylazine (80–100/8–10 mg/kg). There was no procedure-related death. The coccygeal (Co)3/4 and Co5/6 IVDs in each mouse were injured with a 26G needle, while Co4/5 and Co6/7 served as intact controls. Co3/4 (injured) and Co4/5 (intact control) discs were isolated individually for RNA extraction. Co5/6 (injured) and Co6/7 (intact control) motion segments were isolated enbloc for histological examination (the arrow indicates injury to the Co5/6 disc in Figure 1A and Figure 2A). Routine histological preparations include embedding and sectioning, followed by Hematoxylin and Eosin (H&E) and Safranin O staining [23].

**RNA isolation and quantitative Real-Time PCR**. The IVD tissues were dissected, and total cellular RNA was isolated by the Trizol method as described previously [9]. CDNA was synthesized with all RNA from each IVD as described before [23]. The primer sequences for *Gapdh*, *Cxcl1*, and *Adam8* have been published [9]. Primers for *Il6* were purchased (QuantiTect Primer, Qiagen, MD, USA). Real-time PCR was performed, and relative expression was calculated with *Gapdh* as endogenous control [23]. Note that genes and proteins were named according to the “Rules and guidelines for nomenclature of mouse genes” by the International Committee on Standardized Genetic Nomenclature, where murine genes were italicized, with only the first letter capitalized, and proteins were non-italicized, all letters capitalized [25].

**Histological Scoring and Quantification of Safranin O staining.** The paraffin-embedded tissues were sectioned. Serial sagittal sections were stained with H&E or Safranin O and digitized (Figure 1A and Figure 2A). Histological grading was performed according to Melgoza et al. [26], using H&E-stained sections by two clinician-scientists in a double blinded manner. Histological scores ranged from 0–35. Injured and intact discs from mutant and WT mice histological grades were compared using the average scores of the raters. To assess inter-observer variability of the total scores, an intraclass correlation coefficient (ICC) was calculated.

The digitized Safranin O-stained sections were analyzed by ImageJ software (Version 1.54r, NIH Image). Briefly, the IVD regions were cropped (Figure 2A), and percentages of red pixel in the whole color spectrum were calculated as described previously [17].

**Statistical Analysis**. The difference in *Adam8*, *Cxcl1*, *Col1a1*, *Col2a1* and *Gapdh* PCR cycle thresholds (ΔCT) and Safranin O pixel proportion were calculated and logarithmically (log10) transformed for each injured/intact pair to stabilize the variance and normalize the distributions. To assess differences in ΔCT and Safranin O staining between WT and *Adam8^EQ^* genotypes and mouse age, a 2-factor analysis of variance (ANOVA) stratified on intact/injured was performed, where genotype and age were grouping factors. Results of these analyses are presented as log10(ΔCT) or log10(safranin O). For histologic grading, data were also transformed to stabilize the variance and normalize the distribution but using a log10(histology score + 1). Results for histology grading were then transformed [10^(histology score)^ − 1)] to an interpretable scale for presentation purposes. For all analyses, to adjust for multiple pairwise comparisons, post hoc Tukey-Kramer tests were used. A *p* value of <0.05 was considered statistically significant. All analyses were performed using SAS statistical software (Version 9.4, SAS Institute, Cary, NC, USA). Figures were generated using GraphPad Prism (version 10.0.6, GraphPad Software, San Diego, CA, USA).

## 3. Results

### 3.1. Spontaneous Degeneration-like Changes with Aging in Intact IVDs and Moderate Degree of Changes in Injured IVDs of Both WT and Adam8^EQ^ Mice

The scoring was based on H&E-stained sections (Figure 1A). The scores of 2 graders correlated well [n = 47 mice; ICC = 0.9593 for intact, and 0.9353 for injured IVDs]. Intact IVDs of both WT and *Adam8^EQ^* mice scored normal at 3 months of age (range: 0–1.5), while injured IVDs ranged from normal to moderate degeneration-like changes (score range: 1–26). At 10 months of age, most of the intact IVDs of *Adam8^EQ^* and WT mice were normal or mildly degenerative, with only 1 disc scored 14 (moderate) in each mouse type. Injured IVDs of 10-month-old mice scored 5–24. The 18-month-old mice have remarkably well-preserved IVD structure, with scores ranging 0–13 in both *Adam8^EQ^* and WT mice, while the injured IVDs scored 4–22.5. Consistent with previous findings, histological scores are higher in injured IVDs in both WT and *Adam8^EQ^* mice at all ages (*p* < 0.01; Figure 1B).

In WT mice, intact IVDs scored lower at 3 months of age than 10 or 18 months of age (3- vs. 10-month: mean Δ = −3.9, 95% CI: −5.2, −2.9; 3- vs. 18-month: mean Δ = −1.5, 95% CI: −2.1, −1.0, respectively; *p* < 0.01). No significant differences in histological scores were found in injured IVDs of WT mice among different ages (*p* > 0.80; Figure 1B). Similarly, in *Adam8^EQ^* mice, intact IVDs scored lower at 3 months of age than 10 or 18 months of age (3- vs. 10-month: mean Δ = −2.6, 95% CI: −3.0, −2.2; 3- vs. 18-month: mean Δ = −1.4, 95% CI: −2.1, −1.0, respectively; *p* < 0.01). No significant differences in histological scores were detected in intact IVDs of *Adam8^EQ^* mice among various ages (*p* > 0.05). No differences in histological scores between WT and *Adam8^EQ^* mice within intact or injured IVDs were found (*p* > 0.05; Figure 1B).

### 3.2. Safranin O Staining Intensity Decreased with Age in Both Adam8^EQ^ and WT Mice

Safranin O stains proteoglycans red [27]. The injured IVD tissue showed a visible loss of red staining at 1 week post injury in all mice, regardless of age and genotype (Figure 2A).

The intact IVDs in WT 10-month-old mice stained less intensely for Safranin O than in 3-month-old controls (logΔ = 0.96, 95% CI: 0.46, 1.46; *p* < 0.01). Surprisingly, the intact IVDs in 18-month-old mice stained redder than in 10-month-old mice (logΔ = 0.71, 95% CI: 1.22, 0.21; *p* < 0.01), and no differences were found between 3- and 18-month-old intact or injured IVDs (*p* > 0.05; Figure 2B).

In the 10-month-old *Adam8^EQ^* mice, both the intact and injured IVDs stained less intensely for Safranin O than in 3-month-old mice (intact 3- vs. 10-month: logΔ = 0.93, 95% CI: 0.08, 1.78; *p* = 0.03; injured: logΔ = 1.22, 95% CI: 0.35, 2.09; *p* < 0.01). Intact 3-month-old IVDs also stained more strongly than 18-month-old mouse IVDs (logΔ = 0.97, 95% CI: 0.13, 1.80; *p* = 0.02). However, we found no differences among the injured mice of 3 ages (*p* > 0.05). There were no statistically significant differences in % Safranin O staining between the WT and *Adam8^EQ^* mice of the same age in either intact or injured IVDs (*p* > 0.05; Figure 2B).

### 3.3. Differences in Collagen Gene Expression in Adam8^EQ^ and WT Mice of 3 Ages

**Figure 3:**
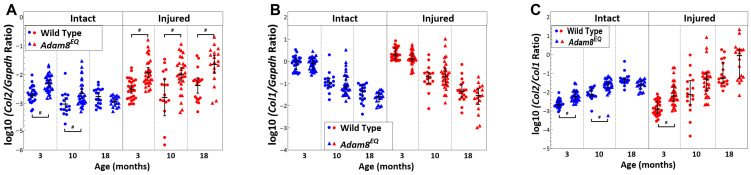
*Col2* and *Col1* Gene Expression in wild type (WT) and Adam8-mutant (*Adam8^EQ^*) Mouse Intervertebral Discs. (**A**) *Col2* gene expression; (**B**) *Col1* gene expression; (**C**) *Col2/Col1* gene expression ratio; each symbol represents data from one mouse [3 months: WT (n = 27), *Adam8^EQ^* (n = 30); 10 months: WT (n = 16), *Adam8^EQ^* (n = 29); 18 months: WT (n = 18), *Adam8^EQ^* (n = 18)]; **error bar**: mean ± 95% confidence interval; ^#^ *p* ≤ 0.01.

At 3 months of age, type II Collagen (*Col2*) gene expression was higher in the *Adam8^EQ^* mice compared with WT in both intact and injured IVDs [intact *Adam8^EQ^* vs. intact WT: logΔ = 0.39; 95% CI: 0.13, 0.64; injured *Adam8^EQ^* vs. injured WT: logΔ = 0.57; 95% CI: 0.08, 1.06; *p* < 0.01]. At 10 months of age, *Col2* gene expression remained higher in both the intact and injured *Adam8^EQ^* IVDs compared with WT [intact *Adam8^EQ^* vs. intact WT: logΔ = 0.37; 95% CI: 0.07, 0.68; injured *Adam8^EQ^* vs. injured WT: logΔ = 0.80; 95% CI: 0.23, 1.37; *p* < 0.01]. At 18 months of age, *Col2* gene expression is still higher in the injured *Adam8^EQ^* mouse IVDs than in the WT IVDs post injury [logΔ = 0.73; 95% CI: 0.12, 1.34; *p* < 0.01], but the difference was not significant in intact IVDs (*p* > 0.05; Figure 3A).

No significant differences in *Col1* gene expression level were found in either the intact or injured *Adam8^EQ^* mouse discs compared with WT controls (*p* > 0.05). There was a progressive decrease in *Col1* gene expression with age, in both the WT and *Adam8^EQ^* mouse IVDs, regardless of the injury status (*p* < 0.01; Figure 3B).

Ratio of *Col2/Col1* gene expression increased progressively with age, related to the progressive decrease in *Col1* gene expression with age in both the intact and injured IVDs (*p* < 0.05). In the intact murine IVDs, similar to the *Col2* gene expression pattern, *Col2/Col1* gene expression ratio was higher in the *Adam8^EQ^* than in the WT mouse at 3 and 10 months of age [logΔ = 0.42; 95% CI: 0.18, 0.65 and logΔ = 0.45; 95% CI: 0.18, 0.73, respectively; *p* < 0.01], but not different in 18-month-old mice [logΔ = 0.01; 95% CI: −0.29, 0.30; *p* = 1.00]. In the injured IVDs, ratio of *Col2/Col1* gene expression between *Adam8^EQ^* and WT mouse IVDs differs only at 3 months of age [logΔ = 0.77; 95% CI: 0.18, 1.35; *p* < 0.01]. At 10 or 18 months of age, *Col2/Col1* gene expression ratio differences between mice of the two genotypes were not statistically significant [logΔ = 0.68; 95% CI: −0.01, 1.37; *p* = 0.05 and logΔ = 0.68; 95% CI: −0.06, 1.42, *p* = 0.09; respectively; Figure 3C].

### 3.4. Differences in Inflammatory Marker Gene Expression Between Adam8^EQ^ and WT in Young Adult Mice Diminish with Age

**Figure 4:**
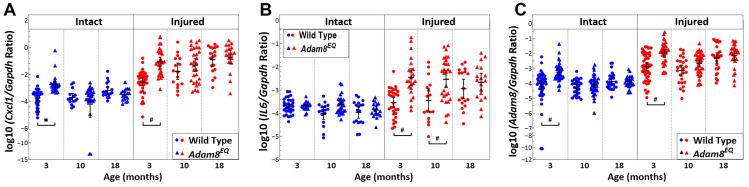
*Il6*, *Cxcl1* and *Adam8* Gene Expression in the Wild Type (WT) and Adam8-mutant (*Adam8^EQ^*) Mouse Intervertebral Discs. (**A**) *Cxcl1* gene expression; (**B**) *Il6* gene expression; (**C**) *Adam8* gene expression; each symbol represents data from one mouse; at 3 months: WT (n = 38), *Adam8^EQ^* (n = 30); 10 months: WT (n = 16), *Adam8^EQ^* (n = 29); 18 months: WT (n = 18), *Adam8^EQ^* (n = 18); **error bar**: mean ± 95% confidence interval; * *p* ≤ 0.05; ^#^ *p* ≤ 0.01.

*Cxcl1* gene was expressed at a higher level in the 3-month-old *Adam8^EQ^* mice than WT mice, in both intact and injured IVDs [intact *Adam8^EQ^* vs. intact WT: logΔ = 1.00; 95% CI: 0.12, 1.88; *p* = 0.02; injured *Adam8^EQ^* vs. injured WT: logΔ = 1.48; 95% CI: 0.76, 2.21, *p* = 0.02]. But there were no significant differences between the mice of the two genotypes at 10 and 18 months of age (*p* > 0.05). Consistent with previous reports, *Cxcl1* gene expression was elevated in the injured compared with intact discs, in both the *Adam8^EQ^* and WT control mice, at all ages (*p* < 0.01; Figure 4A).

*IL6* gene was expressed at a higher level in the injured *Adam8^EQ^* mouse IVDs than WT at 3 and 10 months of age [3-month injured *Adam8^EQ^* vs. WT: logΔ = 1.09; 95% CI: 0.43, 1.76; 10-month injured *Adam8^EQ^* vs. injured WT: logΔ = 0.91; 95% CI: 0.17, 1.65; *p* < 0.01]. However, at 18 months of age, when comparing injured or intact IVDs of *Adam8^EQ^* and WT mice, no significant differences in *IL6* gene expression were detected (*p* > 0.05; Figure 4B).

In the 3-month-old young adults, *Adam8* expression was consistently higher in the *Adam8^EQ^* mice compared with WT controls, in both injured and intact IVDs [intact *Adam8^EQ^* vs. WT: logΔ = 1.20; 95% CI: 0.55, 1.86; injured *Adam8^EQ^* vs. WT: logΔ = 0.79; 95% CI: 0.31, 1.27; *p* < 0.01]. However, at 10 or 18 months of age, no significant differences in *Adam8* gene expression were found in either intact or injured IVDs between mice of the two genotypes (*p* > 0.05; Figure 4C).

## 4. Discussion

Consistent with previous findings by our group and others, IVD degenerates with increased age in the intact mouse spine [14,15,16,17,28]. The injured IVDs of *Adam8^EQ^* mice expressed higher levels of inflammatory genes than controls at 3 months of age [22]. In addition, ADAM8-inactivation seemed to retard IVD degeneration in the intact lumbar IVDs [21]. However, effects of aging when comparing WT and *Adam8^EQ^* mice in the IVD injury model have not been reported previously. The current study compared injury responses between WT and *Adam8^EQ^* mice of different ages, to determine if the inclusion of aged animals was needed.

To our knowledge, this work represents the first direct comparison of histological scores in IVDs from young, middle-aged, and old WT and *Adam8^EQ^* mice, facilitated by a recent consensus on histological evaluation [26]. In response to injury, degenerative features were apparent in IVDs from mice of all ages in both genotypes, but no differences among mice of different ages or genotypes. A less severe injury (e.g., with a thinner needle) might reveal subtle differences in histological scores. In addition, Bhadouria et al. described the biomechanical and histological features of injuries to lumbar spines of 4 to 24 months old mice ex vivo. IVD/endplate injuries caused more severe changes to mouse lumbar IVD biomechanical properties in aged mice than in younger mice [29]. We did not find significant histological differences between injured IVDs from the mutant or WT mice of 3 different ages. It would be a worthwhile future direction to determine the biomechanical properties of injured mouse tail IVDs from mice of various ages to uncover potential differences not revealed by histological scoring.

Percent of red in Safranin O-stained histological sections has been used to quantify proteoglycan content previously [23]. Here, we have shown that 10-month-old intact and injured IVDs stained less red than those of 3-month-old mice, for both WT and *Adam8^EQ^* genotype, suggesting that % Safranin O is a more sensitive marker for disc morphology than histological score. Notably, no additional decrease in Safranin O staining was observed in intact or injured discs of 18-month-old mice compared with 10-month-old animals, irrespective of genotype. One possibility is that only mice with healthy appearing IVDs survived to 18 months, since approximately 20% of animals died of natural causes between age 10 and 18 months.

*Col2* gene expression was consistently higher in the injured *Adam8^EQ^* mouse IVDs than WT mice of all 3 ages, but was higher in intact IVDs of *Adam8^EQ^* than WT mice only at 3 and 10 months of age. *Col1* gene expression decreased progressively with age in both *Adam8^EQ^* and WT controls, but no difference was found between mice of the two genotypes. Given the importance of type II collagen in the IVDs, the elevated *Col2* gene expression could explain, at least in part, the more robust AF in the *Adam8^EQ^* than in WT mice [21,22].

The gene products of *Cxcl1*, *Il6*, and *Adam8* are critical, early molecular markers involved in the inflammatory response, matrix remodeling, and progression of IVD degeneration [23]. In the context of IVD degeneration in response to injury, *Il6* likely mediate acute inflammatory responses, while *Adam8* acts as a key matrix-degrading enzyme and mediator of the chronic phase of degeneration. Following injury to the IVD, *Cxcl1* expression is significantly upregulated, peaking early (e.g., day 1 post-injury in mice) but remaining elevated into the chronic phase (4 weeks), indicating a role in both acute inflammation and the transition to chronic, persistent inflammation [23]. In the human patients, elevated serum CXCL1 levels were correlated to spinal pain [30], genetic variation of IL6 and elevated serum levels have been associated with degenerative disc disease [31,32], and elevated ADAM8 was correlated with IVD degeneration [18]. CXCL1 acts as a major inflammatory cell chemoattractant [33]. While IL6 level in the IVDs is associated with pain and degeneration [34], the data interpretation could be affected by timing of sample collection since it is elevated immediately after injury, and returns to baseline 4 weeks post injury, while *Cxcl1* and *Adam8* gene expression remain elevated compared with the intact IVDs in the mouse IVD injury model [23]. In summary, these 3 genes act in concert to drive degeneration: injury induces *Il6* and *Cxcl1*, initiating inflammation. This leads to the recruitment of immune cells and upregulation of *Adam8*, which then breaks down the structural components (e.g., fibronectin) of the disc, causing a vicious cycle of matrix degradation and further inflammation.

Inflammatory markers (e.g., *Cxcl1*, *Adam8*, and *Il6*) were elevated in mice of both genotypes at all ages post injury. Among these, *Cxcl1* and *Adam8* gene expression in both intact and injured IVDs was higher in the *Adam8^EQ^* mice than WT controls at 3 months of age, but we did not find significant differences between the two genotypes at 18 months. Similarly, *Il6* gene expression was higher in injured IVDs of *Adam8^EQ^* than WT mice at 3 and 10 months of age, but there were no differences at 18 months of age. Taken together, gene expression differences between mice of the 2 genotypes are most readily detectable at 3 months of age.

Sex and genetic background, in addition to age, are important biological determinants of disc degeneration and back pain [35]. Here, mice on the DBA background were used to match the genetic background of *Adam8^EQ^* mice [20,22], with a similar number of male and female mice. No significant differences in histological features and gene expression levels between male and female mice of various ages were found, likely because sex-related differences are subtle and would requires a larger sample number to be detectable.

## 5. Conclusions

Morphological features and gene expression in IVDs of *Adam8^EQ^* and WT mice of 3 ages were directly compared. In intact IVDs, significant differences in inflammatory marker and collagen gene expression, along with some morphological features, were detectable in 3-month-old mice. However, these differences diminish with age and were not detectable at 18 months of age. Consistent with previous findings [17], modest age-related differences were observed in responses to injury suggesting that age-matched mice should be used in future experiments. Three months old mice are superior to those aged 10 or 18 months, if the primary purpose is to identify differences in injury responses between WT and *Adam8^EQ^* mouse IVDs.

## Data Availability

Data supporting reported results can be obtained by submitting a request to Dr. Zhang.

## Abbreviations

The following abbreviations are used in this manuscript:

CXCL1	C-X-C motif chemokine ligand 1
IL6	interleukin 6
ADAM8	a disintegrin and metalloproteinase domain-containing protein 8
GAPDH	Glyceraldehyde-3-phosphate dehydrogenase
COL1a1	type I collagen, alpha1 chain
COlL2a1	type II collagen, alpha1 chain
